# Correction: Stress generation, relaxation and size control in confined tumor growth

**DOI:** 10.1371/journal.pcbi.1010288

**Published:** 2022-06-23

**Authors:** Huaming Yan, Daniel Ramirez-Guerrero, John Lowengrub, Min Wu

The funding statement for this article should read as follows:

“J.L., H.Y. and D.R-G. acknowledge partial funding from the National Science Foundation- Division of Mathematical Sciences (NSF-DMS) grants DMS-1953410, DMS-1714973 and DMS-1763272/Simons Foundation (594598, QN) for the Center for Multiscale Cell Fate Research at UC Irvine. M.W acknowledge partial funding from NSF-DMS-2012330. JL additionally acknowledges partial funding from the National Institutes of Health (NIH) grant 1U54CA217378- 01A1 for a National Center in Cancer Systems Biology at the UC Irvine (also provided support to H.Y.), and from NIH grant P30CA062203 for the Chao Family Comprehensive Cancer Center at UC Irvine. The funders had no role in study design, data collection and analysis, decision to publish, or preparation of the manuscript.”
