# Supplementary material for: Stress generation, relaxation and size control in confined tumor growth
Source: PLoS Comput Biol. 2021 Dec 21;17(12):e1009701. doi: 10.1371/journal.pcbi.1009701 (PMC8726498; doi:10.1371/journal.pcbi.1009701)
Supplement: S3 Text — Parameters for Fig 3. (PDF) [file pcbi.1009701.s003.pdf]

Supplementary Material for
"Stress generation, relaxation and size control in confined tumor growth"

(Dated: December 1, 2021)

3. SUPPLEMENTAL DATA FOR FIG 3

Parameters for Fig 3

$R_0 = 75$	tumor initial radius (in μm)
$L = 40$	diffusional length of nutrient (in μm)
$\beta = 0.6$	relaxation rate
$\lambda_0 = 1.1$	rate of cell volume growth
$\lambda_{A,c} = 0.2$	rate of apoptosis
$\Delta_A = 0.9$	maximum rate of the water flux,
$\gamma_A = 0.1$	sensitivity to the feedback on λ_E
$\gamma_c = 0.1$	sensitivity to the feedback on λ_c
$n = 2$	Hill coefficient of feedback on λ_E
$l = 2$	Hill coefficient of feedback on λ_c
$c_H = 0$	nondimensional shear modulus, free tumor
$c_H = 1.2$	nondimensional shear modulus, 0.7% tumor
$c_H = 3.4$	nondimensional shear modulus, 1.0% tumor
