# Supplementary material for: Stress generation, relaxation and size control in confined tumor growth
Source: PLoS Comput Biol. 2021 Dec 21;17(12):e1009701. doi: 10.1371/journal.pcbi.1009701 (PMC8726498; doi:10.1371/journal.pcbi.1009701)
Supplement: S4 Text — Grid-search parameter optimization, parameters and supporting figures for Fig 4. (PDF) [file pcbi.1009701.s004.pdf]

Supplementary Material for "Stress generation, relaxation and size control in confined tumor growth"

(Dated: December 1, 2021)

4. SUPPLEMENTAL DATA FOR FIG 4

We use the data from [1, 2], where colon carcinoma tumor spheroids containing mouse CT26 cell lines were grown under isotropic compression from an osmotically-induced external pressure. Similar to the previous fitting, we assume that $\bar{p} = 0$ in the free case, and only $\bar{p}$ changes in the compressed case. The parameter combination that minimizes the sum of the l^2-error is:

$$\lambda_0 = 1.1, L = 10, \lambda_{A,c} = 0.05, \Delta_A = 0.3, \gamma_A = 0.1, n = 2, \beta = 0.4$$
$$\bar{p} = 2.1 \text{ for the compressed tumor}$$

Without feedback from mechanical stresses, the parameter combination that minimizes the l^2-error is:

	λ	λ_A	L
Free	1.6	1.2	80
Compressed	1.6	1.1	50

Recall that in Fig 2EF (in the main text), when stresses are released by removing the gels, the tumors tend to recover the growth of the unconstrained spheroid. We report similar observations in the case of osmotic pressure. When the pressure is released [1], growth inhibition is reversed and the tumor tends to regrow to the uncompressed (free) size (Fig 4EF). The parameter combination that minimizes the l^2-error using the data presented in Fig 1 in [1] is:

$$\lambda_0 = 2.1, L = 130, \lambda_{A,0} = 1.4, \Delta_A = 0.4, \gamma_A = 0.2, n = 2, \beta = 1.8$$
$$\bar{p} = 0.3 \text{ for the compressed tumor}$$
$$\text{pressure release at } T = 12$$

We list the fitted parameter values of the data in [1–3] below:

	Description	Figs 2,3 [3]	Fig 4A-4D [1]	Fig 4E-4F [2]
λ_0	rate of cell volume growth (per day)	1.1	1.1	2.1
L	diffusional length of nutrient (μm)	40	10	130
$\lambda_{A,c}$	rate of apoptosis (per day)	0.2	0.05	1.4
Δ_A	maximum rate of the water flux (per day)	0.9	0.3	0.4
γ_A	sensitivity to the feedback on λ_E	0.1	0.1	0.2
n	Hill coefficient of feedback on λ_E	2	2	2
β	relaxation rate (per day)	0.6	0.4	1.8
c_H	nondimensional shear modulus, free tumor	0		
$c_{H,0.7\%}$	nondimensional shear modulus, 0.7% tumor	1.2		
$c_{H,1.0\%}$	nondimensional shear modulus, 1.0% tumor	3.4		
$\bar{p}_f$	nondimensional external pressure, free tumor		0	0
$\bar{p}_c$	nondimensional external pressure, compressed tumor		2.1	0.3

Note that the data in [2] (Fig 4E-4F) is different from that in [1] (Fig 4A-D) and correspondingly the fitted parameters are different. In particular, the tumor spheroids from [2] grow more rapidly at early times than those from [1]. Therefore, the rate of cell volume growth (λ_0), rate of apoptosis ($\lambda_{A,c}$), and diffusional length of nutrient (L) are much larger for Fig 4E-F than those for Fig 4A-D.

In Fig 3 in the main text, we showed that upon gel removal, the spatial distribution of the cell density (ρ_c) reverse to the distribution in the free-boundary case. In the case of isotropic compression from an osmotically-induced external pressure ([1]), the distribution of ρ_c also reverses to that in the free tumor.

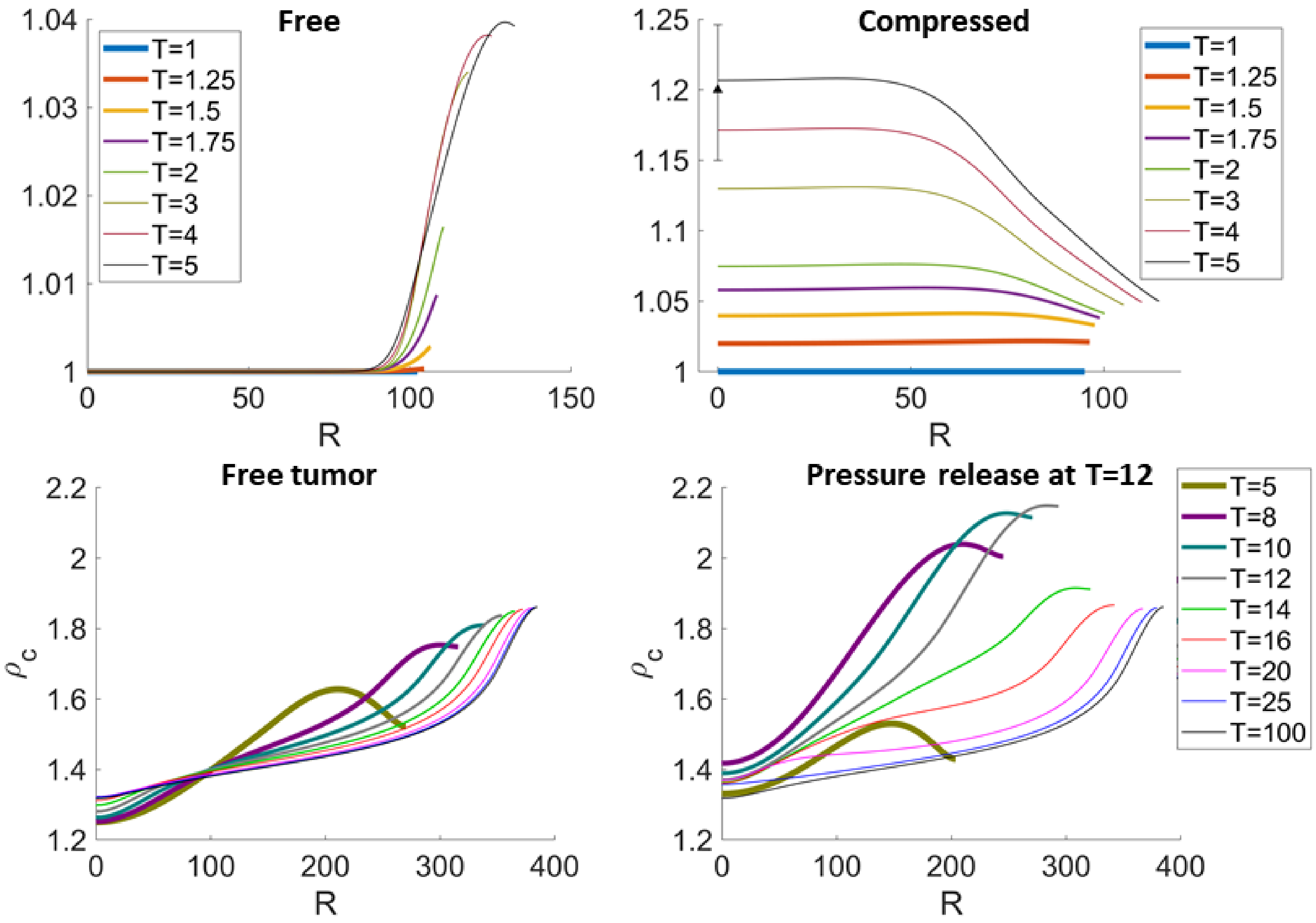

Fig A. Top row: cell densities ρ_c in the free and constrained tumor in Fig 4A. Data in [2] suggests that the cell density at tumor center is approximately 20% larger in the compressed tumor, compared to the free tumor. This is shown by the error bar in the right panel. Similar with Fig 3, the regions with largest cell density shifts towards the tumor center, indicating cells are more packed inside the tumor. Bottom row: after releasing the external pressure, the cell density of the compressed tumor (right panel) recovers to the same level in the free tumor (left panel).

[1] F. Montel, M. Delarue, J. Elgeti, L. Malaquin, M. Basan, T. Risler, B. Cabane, D. Vignjevic, J. Prost, G. Cappello, *et al.*, Stress clamp experiments on multicellular tumor spheroids, Physical review letters **107**, 188102 (2011).

[2] M. Delarue, F. Montel, D. Vignjevic, J. Prost, J.-F. Joanny, and G. Cappello, Compressive stress inhibits proliferation in tumor spheroids through a volume limitation, Biophysical journal **107**, 1821 (2014).

[3] G. Helmlinger, P. A. Netti, H. C. Lichtenbeld, R. J. Melder, and R. K. Jain, Solid stress inhibits the growth of multicellular tumor spheroids, Nature biotechnology **15**, 778 (1997).
