# Supplementary material for: Stress generation, relaxation and size control in confined tumor growth
Source: PLoS Comput Biol. 2021 Dec 21;17(12):e1009701. doi: 10.1371/journal.pcbi.1009701 (PMC8726498; doi:10.1371/journal.pcbi.1009701)
Supplement: S5 Text — Parameters and supporting figures for Fig 5. (PDF) [file pcbi.1009701.s005.pdf]

Supplementary Material for
"Stress generation, relaxation and size control in confined tumor growth"

(Dated: December 1, 2021)

5. SUPPLEMENTAL DATA FOR FIG 5

The effect of β on tumor radius is shown in Fig A. R_∞ is increased by β, which reduces the feedback by reducing the compressive stress at the tumor boundary (see Fig E in S1 Text). When β is small, the tumor radius overshoots before reaching steady state (e.g. blue curve in Fig A), because the circumferential stress reaches steady state faster than the radius. Details of the radii, stresses, elastic energy, and energy density distributions are shown in Fig B-F.

Parameters for Fig 5

$R_0 = 75$	tumor initial radius (in μm)
$L = 40$	diffusional length of nutrient (in μm)
$\lambda_0 = 1.1$	rate of cell volume growth
$\lambda_{A,c} = 0.2$	rate of apoptosis
$\Delta_A = 0.9$	maximum rate of the water flux,
$\gamma_A = 0.1$	sensitivity to the feedback on λ_E
$\gamma_c = 0.1$	sensitivity to the feedback on λ_c
$n = 2$	Hill coefficient of feedback on λ_E
$l = 2$	Hill coefficient of feedback on λ_c

$\beta = 0.05, 0.1, 0.2, 0.4, 0.6, 0.8, 1$ relaxation rate
$c_H = 0, 0.1, 0.2, 0.3, \cdots, 1$ nondimensional shear modulus

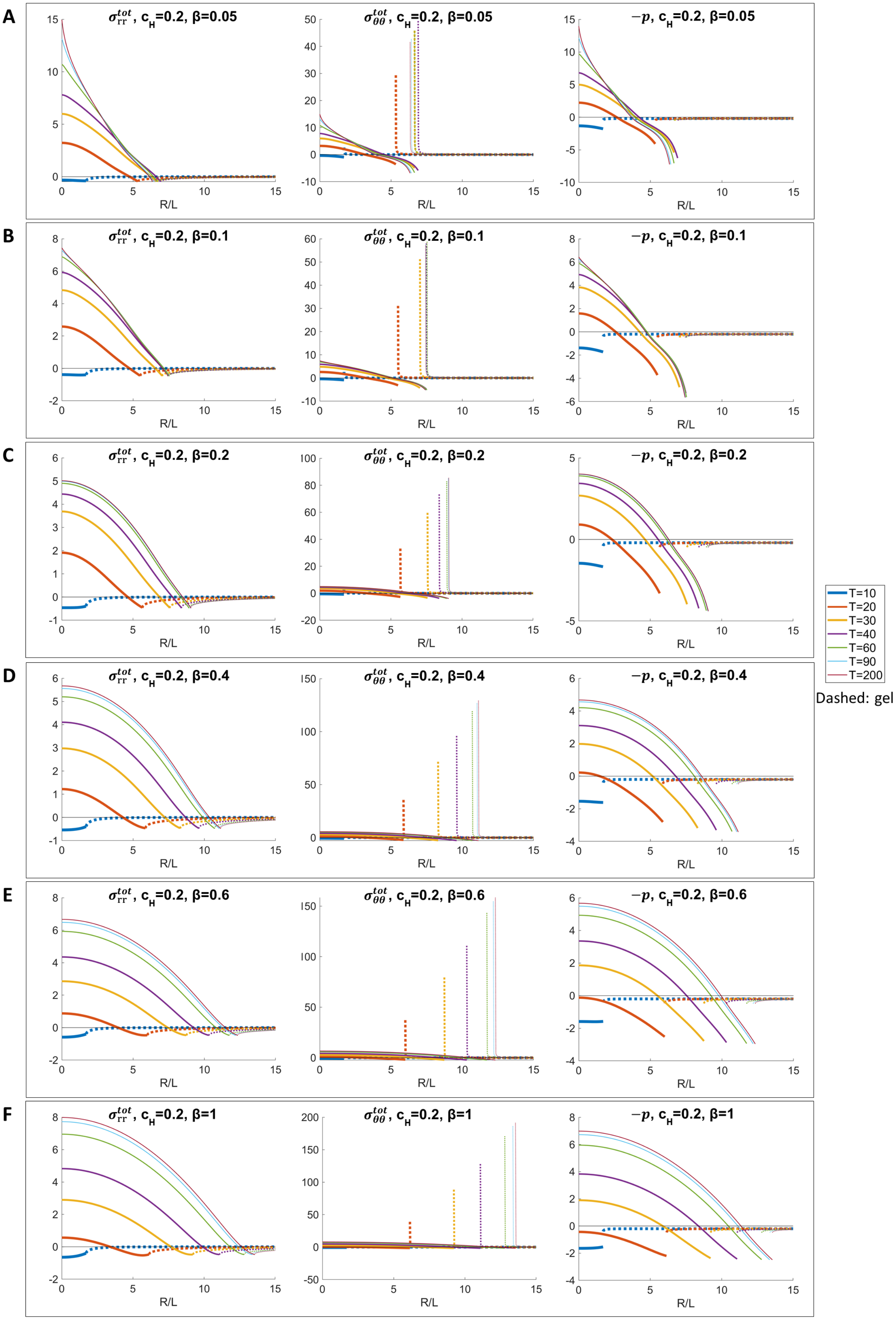

Fig A. Time evolution of the total radial, circumferential stress and pressure inside the tumor (solid lines) and outside (dashed lines), with $c_H = 0.2$ and different β. As β increases from 0.05 to 0.2 (A-C), the magnitude of tensile stress in the tumor is reduced by stress relaxation. When $\beta \geq 0.4$ (D-F), the magnitude of tensile stress increases with β as a size effect of larger tumor.

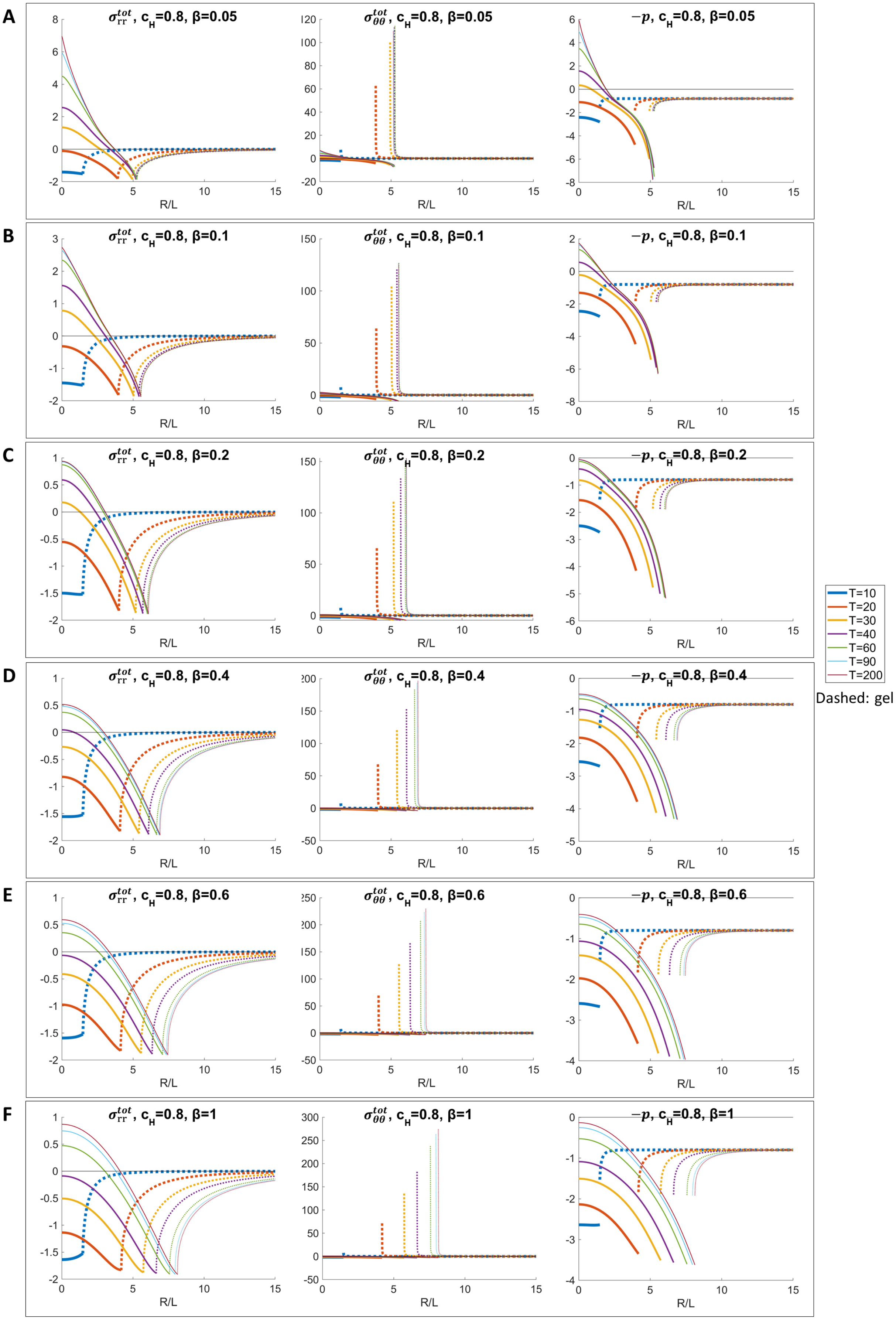

Fig B. Time evolution of the total radial, circumferential stress and pressure inside the tumor (solid lines) and outside (dashed lines), with $c_H = 0.8$ and different β. The tumor is tensile inside when $\beta = 0.05$. The tumor becomes more compressive as β increases. The stress outside the tumor is determined by c_H and R_∞ (Eq. 63 in S1 Text).

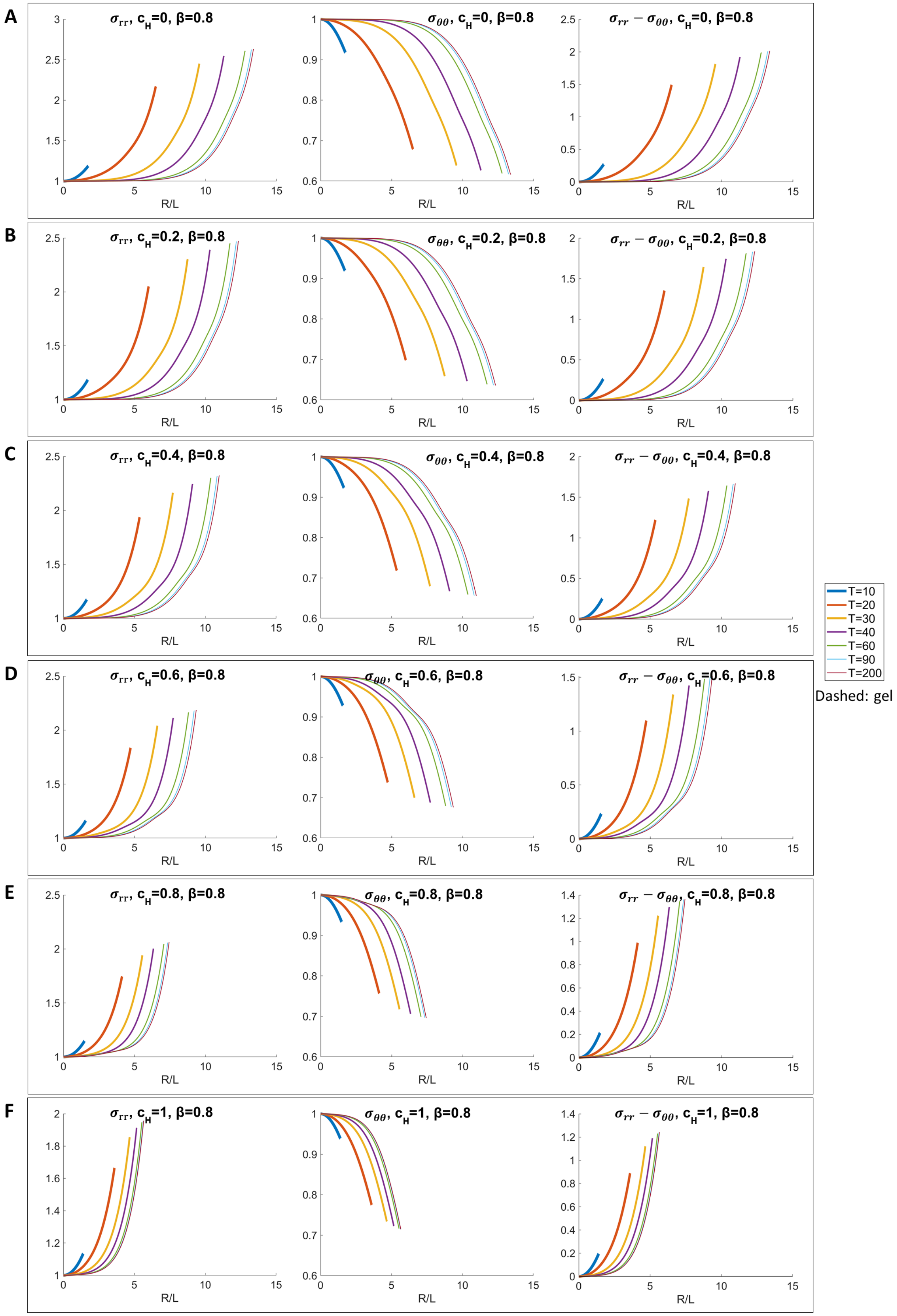

Fig C. Plots of the radial stress (σ_{rr}), circumferential stress ($\sigma_{\theta\theta}$), and stress anisotropy ($\sigma_{rr} - \sigma_{\theta\theta}$) with different c_H. Larger c_H reduces the magnitude and results in more homogeneous stress.

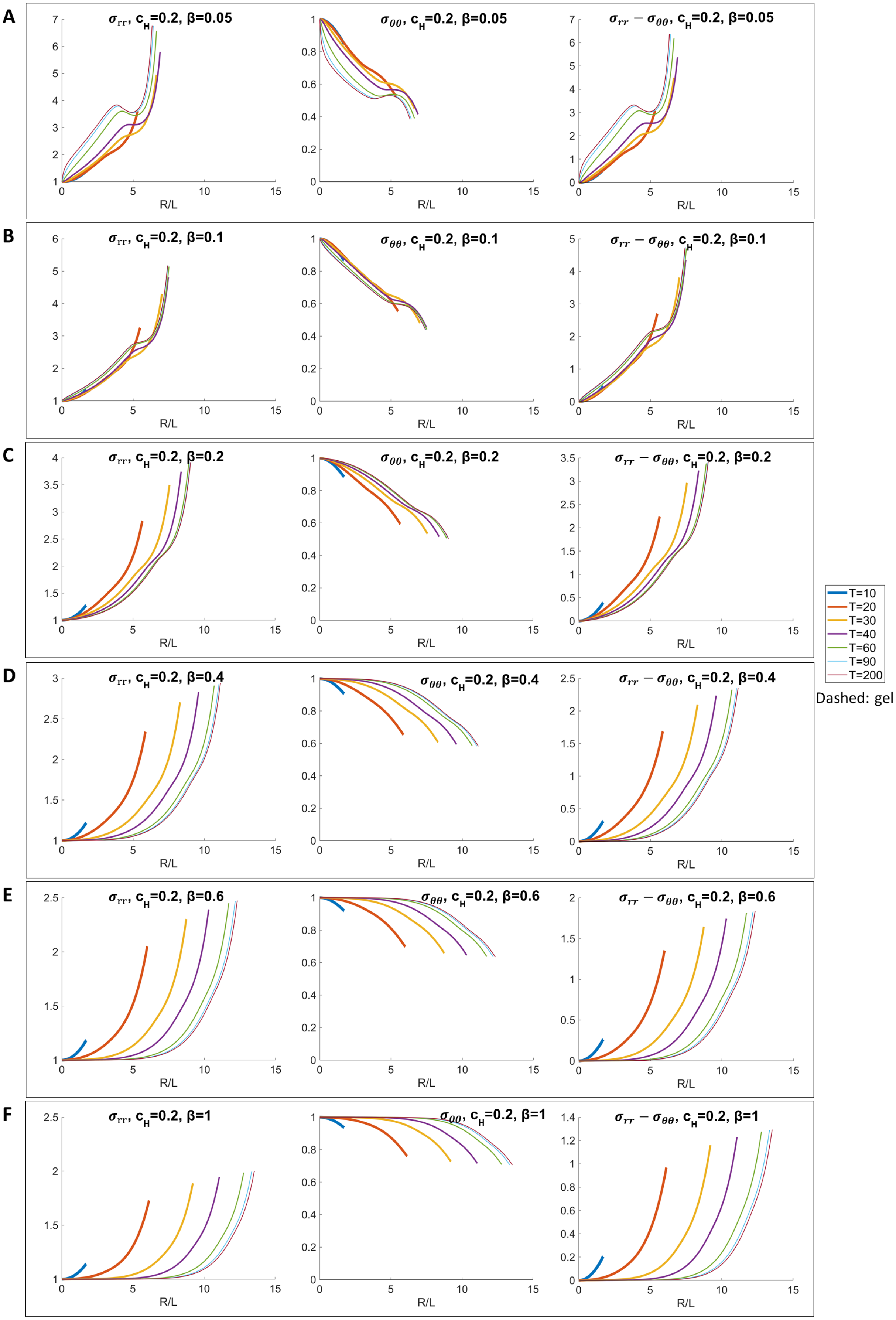

Fig D. Plots of the radial stress (σ_{rr}), circumferential stress ($\sigma_{\theta\theta}$), and stress anisotropy ($\sigma_{rr} - \sigma_{\theta\theta}$) with $c_H = 0.2$ and different β. Larger β changes the shape of stress, reduces the magnitude and results in more homogeneous stress.

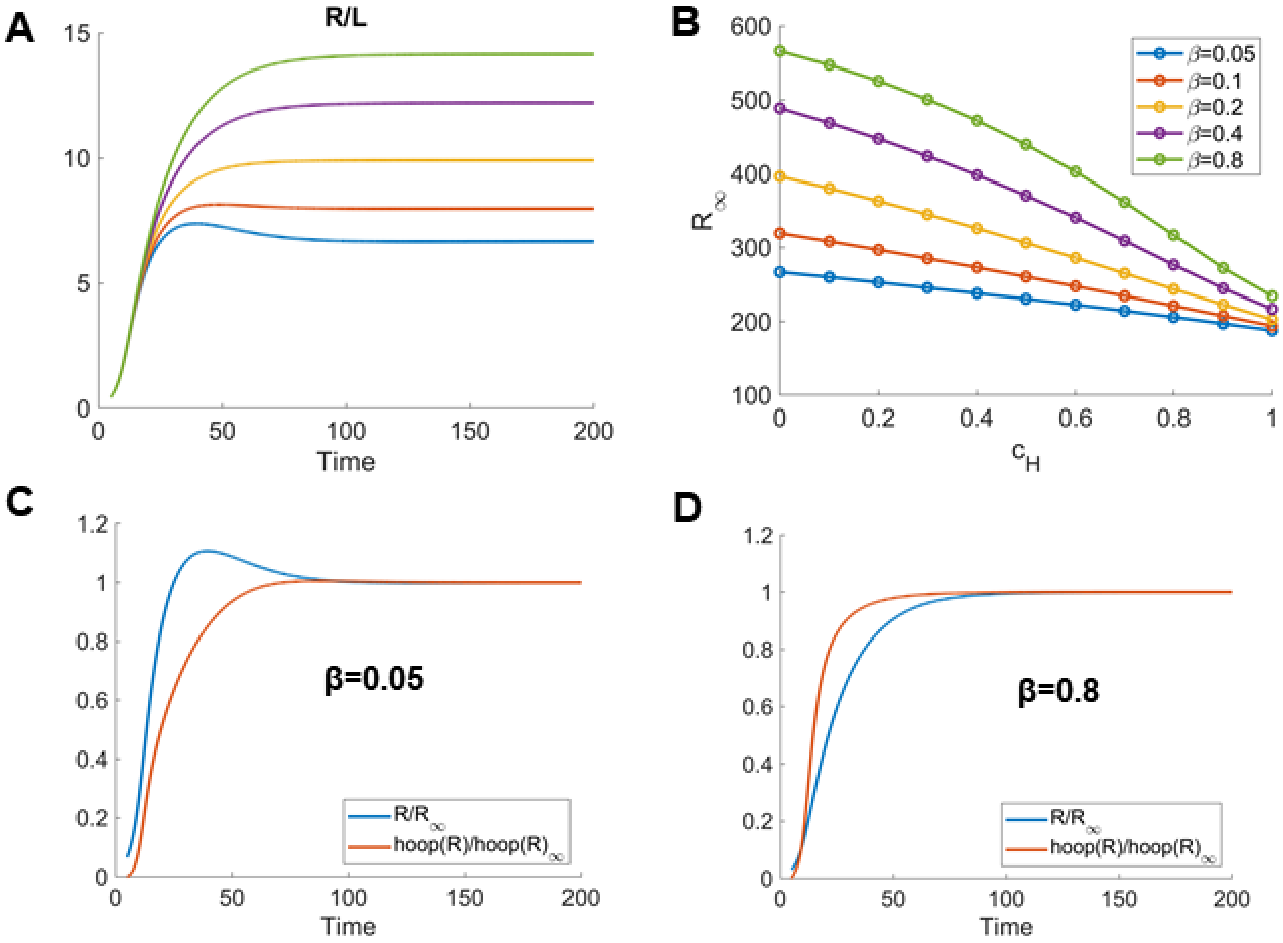

Fig E. (A) Evolution of the tumor radius with $c_H = 0$ and different β. The steady state size is increased by β, which reduces the compressive stress at the tumor boundary. Consequently, the feedback on the apoptosis rate is smaller. When β is small (e.g. 0.05), the tumor radius overshoots before reaching steady state, corresponding to the parameter region marked by the red dashed curves in Fig 5A in the main text. (B) Tumor radius at equilibrium as a function of c_H, for different β. (C, D) The spheroid radius and circumferential (hoop) stress, relative to their values at equilibrium, as a function of time for $\beta = 0.05$ (C) and $\beta = 0.8$ (D), with $c_H = 0$.
